# Targeting the Oncogenic FGF-FGFR Axis in Gastric Carcinogenesis

**DOI:** 10.3390/cells8060637

**Published:** 2019-06-25

**Authors:** Jinglin Zhang, Patrick M. K. Tang, Yuhang Zhou, Alfred S. L. Cheng, Jun Yu, Wei Kang, Ka Fai To

**Affiliations:** 1 Department of Anatomical and Cellular Pathology, State Key Laboratory of Translational Oncology, Prince of Wales Hospital, The Chinese University of Hong Kong, Hong Kong, China; jinglinzhang@cuhk.edu.hk (J.Z.); patrick.tang@cuhk.edu.hk (P.M.K.T.); zyhjoe@gmail.com (Y.Z.); 2 Institute of Digestive Disease, State Key Laboratory of Digestive Disease, The Chinese University of Hong Kong, Hong Kong, China; junyu@cuhk.edu.hk; 3 Li Ka Shing Institute of Health Science, Sir Y.K. Pao Cancer Center, The Chinese University of Hong Kong, Hong Kong, China; 4 School of Biomedical Sciences, The Chinese University of Hong Kong, Hong Kong, China; alfredcheng@cuhk.edu.hk; 5 Department of Medicine and Therapeutics, The Chinese University of Hong Kong, Hong Kong, China

**Keywords:** FGF, FGFR, gastric cancer, monoclonal antibody, small molecule

## Abstract

Gastric cancer (GC) is one of the most wide-spread malignancies in the world. The oncogenic role of signaling of fibroblast growing factors (FGFs) and their receptors (FGFRs) in gastric tumorigenesis has been gradually elucidated by recent studies. The expression pattern and clinical correlations of FGF and FGFR family members have been comprehensively delineated. Among them, FGF18 and FGFR2 demonstrate the most prominent driving role in gastric tumorigenesis with gene amplification or somatic mutations and serve as prognostic biomarkers. FGF-FGFR promotes tumor progression by crosstalking with multiple oncogenic pathways and this provides a rational therapeutic strategy by co-targeting the crosstalks to achieve synergistic effects. In this review, we comprehensively summarize the pathogenic mechanisms of FGF-FGFR signaling in gastric adenocarcinoma together with the current targeted strategies in aberrant FGF-FGFR activated GC cases.

## 1. Introduction

Gastric cancer (GC), the third leading cause of cancer death globally, is considered a heterogeneous disease. Although the prevalence has declined over the past decades, more than half of newly diagnosed cases are found to possess local advancement or metastasis [1,2]. Late diagnosis and lack of effective therapeutics still make GC a challenge globally. For decades, researchers have been dedicated to uncover the mysteries behind GC, not only the medication strategies to alleviate or cure the disease, but the key factors for detecting the challenging disease at its early stage. It has been proven that environmental, etiological, and genetic factors largely contribute to GC development, for example, high salt diets, *H. pylori* infections [3], and *CDH1* mutations [4,5]. Systematically, in-depth and comprehensive mechanistic studies revealed the crosstalk of oncogenic signaling pathways during GC progression as well as pre-cancerous gastric lesion development [6,7,8,9]. Of note, inactivation of the Hippo pathway has been substantially demonstrated in the pathogenesis of GC, via the accumulation of nuclear YAP1 in an uncontrollable manner [10,11,12]. Moreover, recent studies have further uncovered the emerging roles of fibroblast growing factors (FGFs) and their receptors (FGFRs) in the carcinogenesis of some GC subtypes, owing to their molecular characteristics [13]. It has been well documented that the FGF and FGFR families are important regulators for biological development [14,15]. Aberration of FGF-FGFR signaling substantially results in skeletal disorders as well as cancer development, including GC [16]. Since genetic aberrations of FGFR2 have been recently defined, it serves as a diagnostic marker and clinical drug target for GC [17,18,19]. However, development of FGFR2-targeted therapy has been largely decelerated due to recently reported disadvantages. Thus, further investigation of the FGF-FGFR must be continued in order to identify drug targets for GC therapy. This review aims to summarize the updated discoveries and discuss the further prospects of FGF-FGFR signaling in GC pathogenesis and therapy development.

## 2. Emerging Role of FGF-FGFR in Solid Tumors

### 2.1. FGF Family Induces Tumor Growth

FGFRs belong to the receptor tyrosine kinases (RTKs) superfamily. Most of the RTKs are membrane receptors with high affinity to multiple growth factors, cytokines, and hormones, and they contain intracellular domains with tyrosine kinase activity. Canonically, FGFRs are monomers in their inactivation state. Dimerization of the intracellular part occurs after binding with their ligand FGFs. Functional binding of FGF and FGFR leads to cross-phosphorylation and activation of the receptor. Activated FGFRs then transduce biochemical signals into cytosolic activities [20]. Indeed, the FGF family comprises 22 secreted factors that are generally divided into seven subgroups in terms of their phylogenetic relation, homology, and biochemical function [21]. As reported, five FGF subfamilies are released in paracrine and autocrine manners, including FGF1 (FGF1, FGF2), FGF4 (FGF4, FGF5, FGF6), FGF7 (FGF3, FGF7, FGF10, FGF22), FGF8 (FGF8, FGF17, FGF18), and FGF9 (FGF9, FGF16, FGF20). In contrast, the FGF15 (FGF15, FGF19, FGF21, FGF23) subfamily is secreted through endocrine glands as a hormone for metabolic modulation with α- and β-Klotho family proteins. Nevertheless, there are intracellular FGFs (FGF11, FGF12, FGF13, FGF14) that lack secretory N-terminal peptides, which execute their functions independent of FGFRs [22].

FGFs not only show regulatory roles in cell fate and survival, but also exerts biological functions in tissue regeneration and repair [23,24]. In the last few decades, clinical reports have highlighted the importance of FGFs in tumorigenesis, including excessive cell growth and angiogenesis. For example, basic fibroblast growth factor (bFGF) promotes angiogenesis for hepatoma progression [25], and a follow-up study suggested serum bFGF as a biological indicator for invasive and recurrent hepatocellular carcinoma (HCC) [26]. The clinical significance of bFGF was first recognized in patients who received surgical removal of colorectal cancer (CRC) at serological and pathological levels, where expression of bFGF indicated the independency in lymphatic invasion [27]. In addition, *FGF* amplification rated 10% in human malignancies, as overproduction of FGFs enables the communication between epithelial cells and stromal cells in the tumor microenvironment for tumorigenesis [28,29].

### 2.2. FGFR Family Drives Oncogenesis

#### 2.2.1. Functional Structures of FGFR

Interestingly, FGF ligands interact with only four FGFRs (FGFR1–4), which are highly conserved in mammals, although FGFs harbor many family members. In general, FGFRs can be classified into three major domains based on their location relative to the cell membrane: (1) a ternary extracellular immunoglobulin (Ig) (domain I, II, III) that is in charge of binding with ligands; (2) a signal-pass transmembrane helix that acts as a connection; and (3) an intracellular tyrosine kinase (TK) that conveys the signals [30,31]. Generally speaking, the extracellular part of the FGFR provides binding sites for ligand binding, while the intracellular part is responsible for potentiating the relevant signaling pathways. Between the extracellular domains I and II, there is an acidic box region for the FGFR to interact with some molecules other than FGFs, while domains II and III possess the heparin binding site and FGF binding site [32]. The Ig domain III in FGFR1-3 has alternative splicing sites. The domain IIIa remains invariant while the other half varies according to the encoded exon IIIb or IIIc, which are based on tissue-dependent expression [33,34]. This means that the FGFRs only differ between certain parts of the Ig that governs the affinity and specificity of their ligands. There is a single-pass transmembrane domain connecting the Ig domains and the intracellular FGFR domains. The intracellular part of the FGFR includes a juxtamembrane domain for phosphotyrosine binding of adaptors, and two tyrosine kinase (TK) domains. As soon as the TK domains are phosphorylated, the downstream cascades are activated to further expand the signal [35]. Special FGFRs devoid of TK activity, namely FGFR5 or FGFRL1, have been identified and proposed as decoys, interfering with downstream signaling pathways [36]. Due to the diversity of receptor structure and transcript sequence, there are a number of FGFR variants that have been identified. For example, the FGFR2 IIIb isoform has high binding affinity to FGF3, FGF7, and FGF10, while its IIIc form is much preferable to FGF2, FGF4, and FGF20 [20,29]. Further investigation may lead to the discovery of a potential FGFR variant for GC management.

#### 2.2.2. Mechanisms of FGFR in Driving Cancer

Recently, the oncogenic roles of FGFRs have been extensively demonstrated, and somatic alterations and differential expression patterns of FGFR have been seen in different human cancers. Helsten et al. recently depicted a landscape of FGFR aberrations from a large-cohort high-throughput sequencing of cancer patients. In total, FGFR aberrations were detected in 7.1% of the malignancies, including gene amplification (66%), mutations (26%), and rearrangements (8%), suggesting the occurrence of FGFR aberration in most cancer types [37]. Mechanistically, FGFR disorder drives oncogenesis mainly via the following mechanisms: (1) *FGFR* gene amplification: It makes up the majority of the genetic alterations and results in abundant membrane FGFRs, which further augment the activation of its downstream signaling. Gene amplification is common in FGFR1, followed by FGFR2, but rare in FGFR3 and FGFR4. (2) Activating mutations: Most of the mutations exist in the extracellular receptor domains and cause constitutive activation of FGFRs automatically, without the participation of ligands. Activating mutations are frequently found in FGFR2 and FGFR3. (3) FGFRs fusion protein via chromosomal translocation: In this mechanism, the final exon at the C-terminus of the FGFR is replaced by another gene, which results in increasing dimerization and constitutive kinase activity, while ligands are also not required in this manner. (4) Hyperactivation of FGFRs under FGF overproduction from cancer and stromal cells: Additionally, the alternative splicing reconstitutes FGFRs from IIIb to IIIc isoforms, the binding specificity and affinity between FGF and FGFR is altered accordingly. (5) Apart from the genetic alterations of FGF and FGFR, more and more evidence supports that the differential expression of their downstream partners also evidently contributes to the oncogenic progression in multiple cancers [35].

### 2.3. Partner Proteins Mediate FGF-FGFR Signal Transduction

Signal transduction of FGF-FGFR cannot proceed without the participation of partner proteins. Cell adhesion molecules (CAMs), other types of RTKs, and G-Protein-Coupled Receptors (GPCRs) have been found to interact with FGFR family members and regulate a broad range of cell behaviors [38]. Intrinsically, FGFs can be anchored to the extracellular matrix by heparan sulfate proteoglycans (HPSGs) and thus avoid degradation by proteases. FGFs then bind to certain cell-surface FGFRs to form a ternary complex FGF-FGFR-HPSG [39,40]. Otherwise, a deficiency of HPSG results in the enhanced FGF ligand diffusion and failure of the FGF-FGFR signal transduction, which imposes a restriction on cell polarity and motility [41]. As the complex is formed, intracellular tyrosine kinases of FGFR dimerize and cross-phosphorylate on their tyrosine residues at the activation chain. The main intracellular substrates of FGFR are known as phospholipase C (PLCγ) (FRS1), FGFR substrate 2 (FRS2α), and FGFR substrate 3 (FRS2β) [42,43]. These proteins function as adaptors and are directly phosphorylated by the activating FGFRs [44,45]. FRS2 is a lipid-anchored protein and is located on the juxtamembrane domain to recruit signaling components toward the receptor in response to stimulation by ligands [46]. The functional domain of the FRS2 recruits growth factor receptor-bound 2 (GRB2) by four main phosphorylation sites (Tyr196, Tyr306, Tyr349, Tyr392) [47]. GRB2 then enrolls either the guanine nucleotide exchange factor son of sevenless (SOS) or the GRB2-associated binding protein 1 (GAB1) [42]. These proteins form a scaffold for initiating downstream signaling and compose a significant part for signal transduction of the FGF-FGFR signaling. It is noted that some negative regulators exist on the cell surface to counteract the effect of FGFR. One such family is called similar expression to FGF (SEF), members of this family interact with the intracellular domain of FGFRs and inhibit downstream responses. In tumors, the expression of SEF is significantly decreased [48,49].

### 2.4. Signaling Pathways Respond to FGF-FGFR Activation

Upon the recruitment and activation of the FGF-FGFR complex, extracellular signals are turned into intracellular events. Cytosolic signaling pathways aroused by the FGF-FGFR complex are recognized as downstream of FGF-FGFR. It has been well-defined that the Ras-dependent mitogen-activated protein kinase (RAS-MAPK), Ras-independent phosphoinositide 3-kinase (PI3K-Akt), PLCγ-Ca^2+^-PKC, and Janus kinase-signal transducers and activators of transcription (JAK-STAT) act as canonical downstream signaling pathways of FGF-FGFR [50,51,52,53]. On one hand, phosphorylation of FRS2 and GRB2 further initiates the RAS-MAPK and PI3K-AKT signaling pathways by recruiting SOS and GAB1 to the protein complex, respectively. RAS phosphorylates a series of MAPKs such as extracellular signal-regulated kinase 1 (ERK1) and ERK2, which potentiate E26 transformation-specific (ETS) transcription factors to interact and regulate their target genes related to cell proliferation, survival, and transformation [50,54]. As a feedback, inhibitory factors can also be induced by FGF signals. Sprouty (SPRY) interrupts the activation of GRB2, and MAPK phosphatase 3 (MKP3) dephosphorylates ERK1/2 [15]. The PI3K-AKT signaling pathway works differently. After FGF stimulation, GRB2 phosphorylates PI3K-AKT and then inhibits nuclear localization of a pro-apoptotic effector, promoting expression of genes associating with cell survival [55]. In contrast, inhibiting FGFR impairs the function of this pathway and leads to retardation of tumor growth and metastasis [51]. On the other hand, phosphorylation of PLCγ by the FGFR kinase domain hydrolyzes phosphatidylinositol 4,5-bisphosphate to produce inositol triphosphate (IP3) and diacylglycerol (DAG), which support intracellular calcium release and activate protein kinase C (PKC), respectively [56]. Moreover, it has been suggested that amplification of FGFR is required for the signal transducers and activators of transcription-3 (STAT3) activation in cancers. The interaction of FGFR and STAT3 depends on the involvement of JAK [57]. It should be noted that FGF-FGFR signaling cascades also cooperate with other signaling pathways, including Notch [58], Wnt [59], Hedgehog [60], and BMP signaling [61]. Fine-tuning of the cascades ensures homeostasis among normal cells, but their dysfunction may induce multiple diseases and even cancers.

## 3. Deregulation of the FGF-FGFR Signaling in Gastric Carcinogenesis

### 3.1. Significance of FGFR2 in Gastric Tissues

FGF-FGFR signaling exerts multiple biological functions and effects. FGFR2 isoforms IIIb and IIIc are predominantly expressed in the epithelial and mesenchymal tissues [62,63,64]. Along with the understanding of FGFR2, their FGF ligands have been gradually identified. Structurally, FGFR2-IIIb bonds to FGF1, FGF3, FGF7, FGF10, and FGF22 in epithelial tissues; while FGFR2-IIIc responds to a number of FGFs (i.e., FGF1, FGF2, FGF4, FGF5, FGF6, FGF8, FGF9, FGF16, FGF17, FGF18, FGF19, and FGF20) in mesenchymal cells [65,66]. Interestingly, different FGFs will result in various downstream effects via FGFR activation. In gastric tissue, FGFR2 is involved in early epithelial growth before differentiation, and FGF10 and FGFR2-IIIb promote proliferation and patterning of the forestomach. In contrast, silence of both FGF10 and FGFR2 severely induces abnormal lining of gastric epithelium [67].

### 3.2. Aberrant FGF-FGFRs Advance Gastric Tumorigenesis

FGFR2 not only has physiological roles in normal gastric tissue, but also contributes to the development and progression of GC. Indeed, *FGFR2* amplification was detected in GC cells three decades ago [68,69]. The understanding of FGFR2 is extensive, especially in terms of its abnormal genetic alterations that are rare in other FGFR members. *FGFR* amplification is the main genetic alteration in GC, accounting for up to 9% in western populations and 1.2–4.9% in Asian cohorts [13,70]. Nevertheless, mutation and fusion genes are rare in GC patients. From tissue-based studies, incidence of *FGFR2* amplification is equivalent to that of *ERBB2* and *KRAS*, ranging from 2% to 9% according to different methodologies and geographies. Clinical data also manifest that the frequency of *FGFR2* amplification basically contributes to diffuse-type GC [71,72,73,74]. In addition, amplification of *FGFR2* in GC is mutually exclusive with *HER2* and *KRAS* amplification by FISH assay [18,75], suggesting they are independent prognostic biomarkers. Gene amplification is a common cause for mRNA overexpression. In fact, a recent in situ analysis showed that FGFR2 mRNA is highly correlated with *FGFR2* amplification in primary cases clinically, where a high expression level of FGFR2 is associated with poor survival rate of GC patients [76]. Recently, FGFR2 overexpression has been detected in a great portion of GC cases by immunohistochemistry staining, the high level FGFR2-IIIb isoform predicts poor overall survival in patients [19]. A retrospective study revealed that FGFR2 expression was negatively associated with relapse-free survival in a Japanese diffuse-type GC cohort. In that study, although association between FGFR2 expression and survival outcomes in patients with stage II/III GC after surgery and S-1 chemotherapy was insignificant, patients with recurrence after five years of treatment made up a relatively large proportion of the high FGFR2 levels, implying the FGFR2 overexpression may be relevant to GC development [77]. FGFR2 may also contribute to drug resistance of GC. A GC model with *FGFR2* amplification was sensitive to a FGFR inhibitor AZD4547. However, another study questions the efficacy and safety of AZD4547 in GC patients since their progression-free survival rate did not significantly improve with AZD4547 monotherapy compared with paclitaxel, which may due to the intratumor heterogeneity of the *FGFR2* copy-number aberration [78]. Based on these studies, aberrant FGFR2 is largely involved in gastric tumorigenesis and is a candidate to be a diagnostic marker and has the potential to be a therapeutic target for GC treatment. However, challenges will exist until the complexity of the FGFR2 signaling network is resolved.

Autocrine and paracrine FGFs constitute an important functional role in the FGFR2 signaling cascade. In the last two decades, FGF ligands have been reported in multiple cancers, but only a few FGFs were investigated in GC. For example, gastric fibroblast-derived FGF7 increases scirrhous GC cell proliferation in a paracrine manner. Although intrinsic levels of FGF7 are low in GC cells, its corresponding receptor FGFR2 is highly expressed. Subsequently, FGF7 was reported to interact with FGFR2 to promote cell migration and invasion in GC [79,80]. On the other hand, a study found that FGF9 triggers proliferation and inhibits apoptosis of GC cells in an autocrine manner in a Chinese GC cohort [81]. At the genetic level, amplification of *FGF* genes may lead to their overproduction in GC, specifically, *FGF10* amplification has been reported in 3% of GC and in 5.7% of gastric adenocarcinomas [82,83]. FGF10 is correlated to GC cell invasion and has been suggested as a prognostic biomarker and potential drug target in gastric adenocarcinoma [84]. In our recent study, we explored the FGF mRNA profiling in 10 GC cell lines by microarray analysis, where FGF18 showed the highest expression among all the FGF members. This study also identified clinical correlation of FGF18 and highlighted FGF18 as a potent diagnostic indicator in GC. Upon FGF18 stimulation, cell growth is facilitated by activation of SMAD2/3 and suppression of ATM signaling [85]. Nevertheless, the molecular network of FGF-FGFRs responsible for GC progression remains to be revealed.

### 3.3. FGFR2 Crosstalk in GC

It is believed that the FGFR2 aberration fundamentally contributes to GC development, but how FGFR2 coordinates with other regulatory signaling remains unclear. Investigations of FGFR2 and other oncogenic signaling have been conducted to decipher the comprehensive network.

The amplification of FGFR2 has been implied to facilitate cell growth in GC through crosstalking with other RTKs. It is reported that activated epidermal growth factor receptor (EGFR), human epidermal growth factor receptor 3 (HER3), and MET correlate with drug hyposensitivity of GC cells with *FGFR2* amplification. Interestingly, a combination of an FGFR2 inhibitor and EGFR neutralizing antibody partially enhanced drug sensitivity of GC in vitro and in vivo, suggesting these RTKs may cause drug resistance in cancer cells under FGFR2 inhibition. Eventually, a novel mechanism was identified whereby RTKs can coexpress with FGFR2 and synergistically promote the growth of GC [86]. In contrast, another study reported that HER2, MET, and FGFR2 are mutually exclusive oncogenic drivers, where a large number of HER2-negative patients were highly sensitive to the MET- and FGFR2-targeted therapies [87]. However, these contradictory conclusions may be attributable to the differences of the GC cohorts and the experimental models applied in the studies. One possible reason is that the former study focused on *FGFR2* amplification cases where patients were hyposensitive to AZD4547, while the later one concerned both gene amplification and overexpression. Nevertheless, these results examined the potential relationship between FGFR2 and other RTKs, though the underlying molecular mechanisms are not fully understood. Combined therapy for targeting both FGFR2 and RTKs may be a new strategy for clinically treating GC.

**Figure 1:**
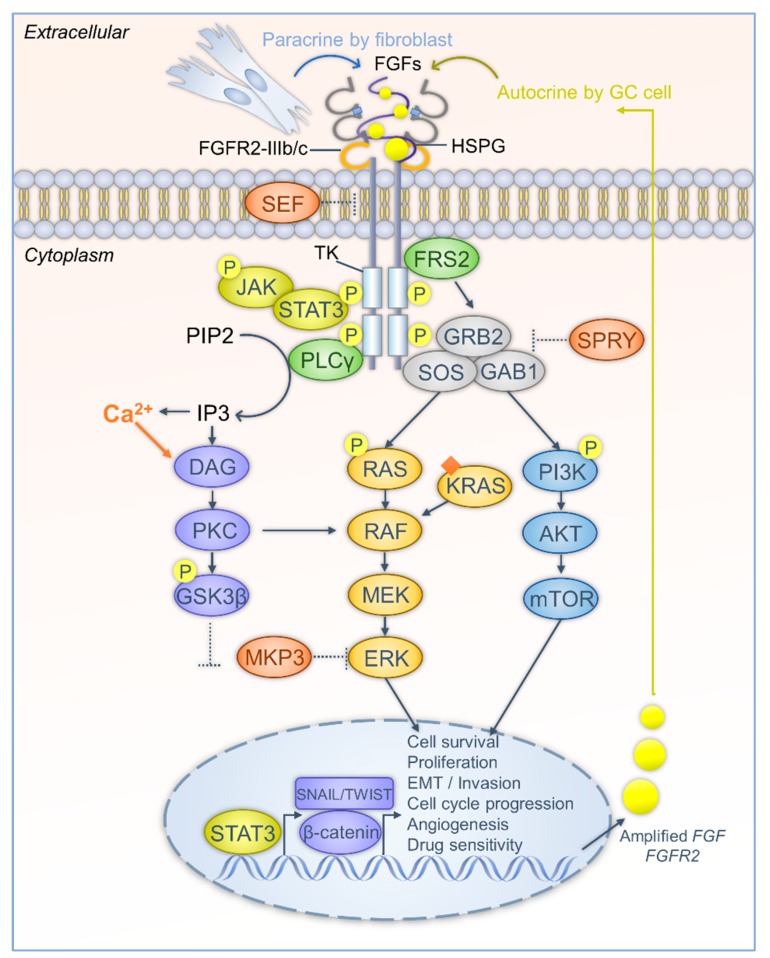
The FGF-FGFR cascade interplays with the downstream signaling network in GC progression. Firstly, FGFR is aberrantly activated in GC cells. FGFs can be released mainly in two ways, in a paracrine manner by gastric fibroblasts, and in an autocrine manner from cancer cells. Gene amplification of *FGFs* and *FGFRs* leads to overproduction of FGFs and FGFRs. FGFs are stabilized and bind to FGFR via HSPG. Alternative splicing of FGFR induces two isoforms that highly are expressed in GC. The isoforms show different affinity to FGFs and contribute to diverse cellular processes. The intracellular region of the FGFR has tyrosine kinase (TK) activity. FGF stimulation leads to dimerization, phosphorylation, and activation of FGFR. The inhibitory effect of SEF is attenuated in GC cells. Secondly, after FGFR activation, adaptor proteins are recruited and also activated by phosphorylation. FRS2 further recruits GRB2, GAB1, and SOS to form a complex. The complex activates RAS-MAPK and PI3K-Akt-mTOR signaling pathways and transduces FGF stimulation into transcriptional regulation to forward tumorigenesis. The inhibitory effects of SPRY and MKP3 are abrogated in GC cells. PLCγ hydrolyzes PIP2 to IP3, increases Ca^2+^ levels, triggers DAG-PKC signaling, and phosphorylates GSK3β. Then, GSK3β is decreased and β-catenin is released to the nuclei. β-catenin and other EMT transcription factors, SNAIL and TWIST, initiate expression of oncogenes that are required for GC progression. Besides, JAK-STAT3 is activated by FGFR and contributes to transcriptional regulation of GC progression. (Arrows represent the activation or release routes; dash dots indicate the weakening of inhibitory effects).

In addition, several signaling pathways have been highlighted as downstream of FGFR2 that may also be involved in GC development (Figure 1). Lau et al. revealed a survival mechanism for developing acquired-resistance under FGFR inhibition. They established drug resistance on both primary and patient derived xenograft (PDX) models of various GCs with different *FGFR2* amplification levels by applying FGFR2 inhibitors. Interestingly, they observed that MAPK and AKT signaling pathways were dispensable for drug resistance, but the constitutive inhibition of GSK3β, which depends on activation of PKC, was required for cell survival [88]. Therefore, the FGFR2-PKC-GSK3β axis is considered as the main mechanism causing resistance in GC during anti-FGFR2 therapy. Additionally, PI3K-Akt-mTOR signaling contributes to the oncogenic activity of FGFR signaling in GC. Huang et al. recently suggested that FGFR2 signaling promotes GC by regulating the expression of Thrombospondin 1 (THBS1) and THBS4 via the PI3K-Akt-mTOR pathway. They indicated that FGF7-FGFR2 signaling upregulates THBS1, while THBS4 is decreased by the FGFR2-Akt cascade [80,89]. These studies established that PI3K-Akt signaling partially contributes to the tumor-promoting function of FGFR2 in GC, although the contribution of the THBS family to GC is still not fully understood. Therefore, further studies are required to reveal the detailed mechanisms. Moreover, epithelial mesenchymal transition (EMT) is a well-known mechanism that facilitates tumor cell transformation and distant metastasis during oncogenic progression. FGF-FGFR signaling has been shown to potentiate EMT [20]. The basic components of EMT, WNT signaling, and Twist-related protein 1 (Twist1) have been found to upregulate FGFR2 in GC cell lines. In turn, FGFR2 further amplifies Twist1 mediated EMT and cell invasion, implying dual inhibition of these pathways is needed for GC therapy [90]. Of note, under the FGFR2 signaling cascade, nuclear accumulation of β-catenin and EMT transcription factors, such as SNAIL, have also been proposed [91].

Current findings have uncovered the complicated interactions between FGFR2 signaling and other RTKs and oncogenic signaling pathways in GC. These signaling networks trigger primary and secondary resistance of GC cells under treatment and eventually lead to the advanced stage of disease. Fortunately, a better understanding of the FGFR signaling network will gradually help in the development of novel therapeutic options for GC.

## 4. Targeting Aberrant FGF-FGFR Activation in GC by Specific Antibodies or Small Molecules

As the FGF-FGFR singling plays an oncogenic role in tumorigenesis by crosstalking with or regulating multiple crucial other pathways, targeting of FGF-FGFR by specifically designed therapeutic agents has shed light on the precision of medicine [92]. These agents include specific anti-FGFR monoclonal antibodies, FGF traps [93], non-selective RTK inhibitors, and selective RTK inhibitors.

### 4.1. Specific Antibodies and FGF Traps

In the aberrant FGF-FGFR-activation GC cases, anti-FGF (FGF traps) or anti-FGFR monoclonal antibodies might exert anti-cancer effects for the treatment (Table 1). Compared with tyrosine kinase inhibitors, the specific antibodies targeting FGFs or FGFRs have more specificity and less toxicity because they can avoid the off-target effects.

The specific monoclonal antibodies generated and effectively employed for targeting FGFRs in GC research are quite limited [22]. They include GAL-FR21 and GAL-FR22 antibodies. GAL-FR21 binds only the FGFR2IIIb isoform, whereas GAL-FR22 and GAL-FR23 can directly bind to both the FGFR2IIIb and FGFR2IIIc isoforms, with binding regions respectively in the D3, D2-D3, and D1 domains of FGFR2. GAL-FR21 and GAL-FR22 block the binding of FGF2, FGF7, and FGF10 to FGFR2IIIb. GAL-FR21 inhibits FGF2- and FGF7-induced phosphorylation of FGFR2, and both antibodies dramatically down-modulate the activation of FGFR2 in SNU16 cells (with FGFR2 amplification). These monoclonal antibodies also effectively inhibit the tumor growth of established SNU16 and OCUM-2M xenografts in mice [94]. Another FGFR2b-specific antibody, FPA144, can not only treat GC patients with FGFR2 amplification, but also patients with FGFR2b overexpression who lack FGFR2 gene amplification. FPA144 is still being evaluated in a phase III clinical trial of GC. Another novel antibody-drug conjugate (ADC), namely BAY 1179470, provides preclinical efficacy. It consists of a fully human FGFR2 monoclonal antibody, which binds to the FGFR2 isoforms FGFR2-IIIb and FGFR2-IIIc, conjugated through a noncleavable linker to a novel derivative of the microtubule-disrupting cytotoxic drug auristatin (FGFR2-ADC). Functional studies demonstrated that FGFR2-ADC administration leads to a significant tumor growth inhibition or tumor regression of cell line-based or patient-derived xenograft models of human gastric or breast cancer. Similar to FPA144, FGFR2 amplification or mRNA overexpression predicted high response to BAY 1179470 treatment [95].

As some FGF members, such as FGF18, are abundant in gastric carcinogenesis, using FGF ligand traps is another strategy to neutralize FGF and quench malignancies [85]. An FGF “ligand trap” is comprised of a fusion protein of an immunoglobulin Fc fragment and a soluble FGFR extracellular domain that competitively binds with FGF1, 2, 3, 7, and 10 to suppress ligand-dependent FGFR signaling [93]. For example, the FGF traps FP-1039 (GSK3052230) and sFGFR3 are soluble proteins that contain the extracellular regions of FGFR1 and FGFR3, respectively [96], thus they can successfully neutralize the oncogenic role of FGFs. Another good example is NSC12, acting as an extracellular FGF trap. It can be employed in anti-angiogenic and anti-vascular endothelial growth factor therapy as an FGF antagonist [97].

### 4.2. Small Molecules: Non-Selective and Selective FGFR Inhibitors

Apart from the antibodies or traps, small molecules can also generally and effectively inhibit tyrosine kinase receptor-related signaling (non-selective FGFR inhibitors). SOMCL-085 is a novel FGFR-dominant multi-target kinase inhibitor. This compound can simultaneously inhibit the angiogenesis kinases such as vascular endothelial growth factor receptor (VEGFR) and platelet-derived growth factor receptor (PDGFR). SOMCL-085 potently inhibits FGFR1, FGFR2, and FGFR3 kinase activity, with IC_50_ values of 1.8, 1.9, and 6.9 nmol/L, respectively [99]. In the FGFR1-amplified lung cancer cell line H1581-xenograft mice and FGFR2-amplified GC cell line SNU16-xenograft mice, oral administration of SOMCL-085 for 21 days substantially inhibited tumor growth without loss of body weight. Nintedanib, a triple-angiokinase inhibitor, is a potent and selective inhibitor for tumor angiogenesis through the blocking of the tyrosine kinase activities of VEGFR1-3, PDGFR-alpha and -beta, together with FGFR1-3 [100]. In combination with docetaxel, nintedanib has been approved for the second-line treatment of adenocarcinoma non-small cell lung cancer (NSCLC). In human GC cell lines driven by an FGFR2 amplification, such as KatoIII, nintedanib is also confirmed to be highly effective. Regorafenib has also demonstrated survival benefits in patients with metastatic colorectal and gastrointestinal stromal tumors. More importantly, FGFR2 amplification was the only genetic alteration associated with in vitro sensitivity to regorafenib. Regorafenib induces G1 phase cell cycle arrest in SNU16 and KATOIII GC cells and suppresses their xenograft formation abilities [101]. S49076 is a novel and potent inhibitor of MET, AXL/MER, and FGFR1/2/3. S49076 potentially blocks cellular phosphorylation of MET, AXL, and FGFRs and inhibits downstream signaling pathways in vitro and in vivo. S49076 alone can cause tumor growth arrest in bevacizumab-resistant cancer cells. Based on the favorable and novel pharmacologic profile of S49076, a phase I study is currently being conducted in patients with advanced solid tumors [102]. Ponatinib (AP24534), an oral multitargeted tyrosine kinase inhibitor, has been explored in a pivotal phase II trial in patients with chronic myelogenous leukemia due to its potent ability against BCR-ABL. It has also been shown to inhibit the in vitro kinase activity of all four FGFRs. In a panel of 14 cell lines representing multiple tumor types (endometrial, bladder, gastric, breast, lung, and colon) and containing FGFRs dysregulated by amplification, overexpression, or mutation, ponatinib inhibited FGFR-mediated signaling with IC_50_ values below 40 nmol/L, supporting it as a potent pan-FGFR inhibitor in patients with FGFR-driven cancers [103].

To avoid the off-target effects of non-selective inhibitors, novel selective FGFR inhibitors were generated and employed for specifically blocking the FGF-FGFR cascade in GC. Among all the selective FGFR inhibitors, AZD4547 is the most famous [17]. It is a selective FGFR1, 2, 3 tyrosine kinase inhibitor with potent preclinical activity in FGFR2-amplified gastric adenocarcinoma SNU16 and SGC083 xenograft animal models, together with the patient-derived cells (PDCs) [104]. The randomized phase II SHINE study (NCT01457846) investigated whether AZD4547 improved clinical outcome versus paclitaxel as a second-line treatment in patients with advanced gastric adenocarcinoma displaying FGFR2 polysomy or gene amplification detected by fluorescence in situ hybridization (FISH). However, the final results indicated AZD4547 failed to significantly improve progression-free survival compared with paclitaxel in GC patients with FGFR2 amplification or polysomy [78]. The related molecular mechanism needs to be further addressed. LY2874455, a potent oral selective pan-FGFR inhibitor, was investigated for its efficacy in a phase I clinical trial. LY2874455 was gradually absorbed and generally showed linear pharmacokinetics. The effective half-life span was approximately 12 h. In 15 GC patients, one patient had a partial response, while 12 patients had stable disease. Thus, LY2874455 has a recommended phase II dosing of 16 mg BID in solid-organ cancer patients [105]. However, in FGFR2-amplified GC patients, some will eventually develop an acquired LY2874455 resistance due to a novel FGFR2-ACSL5 fusion protein that is formed [106]. Based on the structure, medicinal chemistry optimization, and unique ADME assays of a covalent drug discovery program, a novel compound, namely PRN1371, was discovered to serve as a highly selective and potent FGFR1-4 inhibitor [107]. In combination with the de novo synthesis program ‘SYNOPSIS’ to generate high scoring and synthetically accessible compounds, alofanib (RPT835) was found to be an effective inhibitor of the FGF/FGFR2 pathway. RPT835 potently inhibited growth of KATOIII GC cells with a GI_50_ value of 10 nmol/L [108]. ARQ 087 is a novel, ATP competitive, small molecule, multi-kinase inhibitor with potent in vitro and in vivo activity against FGFR-addicted cell lines and tumors. It exhibited IC_50_ values of 1.8 nM for FGFR2, and 4.5 nM for FGFR1 and 3. ARQ 087 has anti-proliferative activity in cell lines driven by FGFR dysregulation, including amplifications, fusions, and mutations, such as the SNU16 cell line. It is currently being investigated in a phase I/II clinical trial [109]. BGJ398, a pan-FGFR inhibitor, was also investigated in a GC model. In vitro, FGFR inhibition was most effective in KKLS cells (high FGFR1, FGFR2IIIc, no FGFR2IIIb expression) with inhibition of growth and motility. BGJ398 also showed partial activity in MKN45 cells (intermediate FGFR1, high FGFR2IIIb, low FGFR2IIIc expression), while TMK-1 cells (low FGFR1, no FGFR2IIIb and FGFR2IIIc expression) showed a negative response to this drug [110]. Some of the non-selective and selective FGFR inhibitors that have been investigated in gastric adenocarcinoma are listed in Table 2.

## 5. Conclusions and Future Directions

Although we have made great progress in understanding the molecular mechanisms and crosstalk of FGF-FGFR in gastric carcinogenesis, and are even trying to employ small molecules or specific antibodies to block the oncogenic-driven role of FGF-FGFR signaling, several important issues need to be addressed urgently in future studies. First of all, GC can be subgrouped into intestinal and diffuse type from the histological classification, and it can also be stratified as four molecular subtypes according to TCGA molecular classification, Epstein-Barr virus (EBV)-positive tumors, microsatellite instable (MSI) tumors, genomically stable (GS) tumors, and tumors with chromosomal instability (CIN) [13]. Each subtype has its distinct molecular features and the etiology together with pathological processes are quite different among the subtypes. Thus, we need to re-evaluate the genetic and epigenetic changes and clinical correlations in a large cohort of FGF-FGFR for each subgroup to confirm the impact of different genetic backgrounds on FGF-FGFR activation. For example, in a small size cohort study, high FGFR4 expression correlated with tumor progression and survival in both diffuse and intestinal GC, whereas high expression of FGFR1 and 2 correlated with tumor progression and survival only in diffuse type GC [111]. Secondly, as FGF-FGFR crosstalks with multiple signaling pathways, such as the RAS-MAPK pathway, PI3K-Akt-mTOR pathway [112], and PKC-GSK3β pathway, we need to stratify our primary samples again according to different crosstalks by the immunohistochemistry method combined with FISH analysis. We will re-evaluate the clinical significance and perform co-administration of multiple anti-cancer drugs to achieve synergistic effects. The successful development of highly specific anti-FGFR personalized strategies will rely on our deeper knowledge of the key alterations that drive oncogenesis in GC [113]. Based on the identification of novel key downstream effectors of the FGF-FGFR cascade in gastric carcinogenesis, we aim to effectively and accurately target FGFR-related signaling in this precision medicine era.

## Figures and Tables

**Table 1:** A list of anti-FGFR monoclonal antibodies and FGF traps potentially employed in GC.

Monoclonal Antibodies	Targets	References
GAL-FR21 and GAL-FR22	FGFR2	[94]
FPA144 (Bemarituzumab)	FGFR2 amplification or overexpression	[98]
BAY 1179470	FGFR2 amplification or overexpression	[95]
FGF traps		
GSK3052230	FGFs	[93,96]
NSC12	FGFs	[97]

**Table 2:** The list of non-selective and selective FGFR tyrosine kinase inhibitors reported in GC.

Non-Selective FGFR Inhibitors	Main Targets	References
SOMCL-085	FGFR, VEGFR, and PDGFR	[99]
Nintedanib	FGFR, VEGFR, and PDGFR	[100]
Regorafenib	FGFR2, VEGFR1-3, PDGFR, c-Kit, and RET	[101]
S49076	MET, AXL/MER, and FGFR1-3	[102]
Ponatinib	BCR-ABL, VEGFR2-3, and FGFR1-4	[103]
Selective FGFR inhibitors		
AZD4547	FGFR1, FGFR2 and FGFR3	[17,78]
LY2874455	FGFR1, FGFR2, FGFR3 and FGFR4	[105]
PRN1371	FGFR1, FGFR2, FGFR3 and FGFR4	[107]
RPT835	FGFR2	[108]
ARQ 087	FGFR1, FGFR2 and FGFR3	[109]
BGJ398	FGFR1, FGFR2 and FGFR3	[110]
